# Model-Based Design of Sustained-Release Formulations of Anti-TNF-α Monoclonal Antibodies for Intravitreal Administration

**DOI:** 10.3390/pharmaceutics18040445

**Published:** 2026-04-04

**Authors:** Javier Reig-López, Marina Cuquerella-Gilabert, Javier Zarzoso-Foj, Víctor Mangas-Sanjuán, Virginia Merino, Matilde Merino-Sanjuán

**Affiliations:** 1 Department of Pharmacy and Pharmaceutical Technology and Parasitology, University of Valencia, 46100 Valencia, Spain; javier.reig@uv.es (J.R.-L.); marina.cuquerella@uv.es (M.C.-G.); javier.zarzoso@uv.es (J.Z.-F.); virginia.merino@uv.es (V.M.); matilde.merino@uv.es (M.M.-S.); 2 Interuniversity Research Institute for Molecular Recognition and Technological Development, University of Valencia-Polytechnic University of Valencia, 46022 Valencia, Spain

**Keywords:** uveitis, intravitreal, anti-TNF-α, NAM, PBPK

## Abstract

**Background/Objectives**: While intravitreal administration allows for increased ocular exposure to anti-TNF-α monoclonal antibodies, there is still a need for developing delivery systems able to prolong ocular drug exposure and alleviate patient compliance and safety concerns because of repeated injections. Therefore, the objective of this work was to guide the design of sustained-release formulations of anti-TNF-α monoclonal antibodies for intravitreal administration through a model-based strategy in non-infectious uveitis in the preclinical setting. **Methods**: Using an in-house-developed anterior uveitis disease model in rats, an intravenous reference dose reducing free TNF-α by 90% at the biophase was established. Intravitreal administrations of sustained-release formulations every 24 weeks were then simulated for adalimumab, golimumab and infliximab to evaluate TNF-α kinetics in the anterior chamber of the eye at different release rates. The selected sustained-release formulation was further evaluated for possible formulation issues causing device emptying before the next administration. **Results**: Intravitreal administration of sustained-release formulations releasing adalimumab, golimumab or infliximab at 1.802, 0.979 and 1.442 μg/week, respectively, met the predefined criteria of ≥90% reduction in free TNF-α at the biophase. TNF-α levels in aqueous humour were anticipated to be the most sensitive to detect possible formulation issues. Formulation emptying 10, 4 or 8 weeks for adalimumab, golimumab and infliximab, respectively, before next administration triggered TNF-α reaching pathological levels at week 24 post-dose. **Conclusions**: This work underscores the potential of new approach methodologies in the preclinical drug development of sustained-release formulations for intravitreal administration in ocular inflammatory disorders with less animal testing and without compromising the accuracy of model-informed predictions for human translation.

## 1. Introduction

Inflammation of the iris or the *corpus ciliare* (iritis or anterior uveitis) is frequent among patients with systemic inflammatory diseases [1]. Different cytokines have been suggested to be involved in the pathogenesis of intraocular inflammation, and among them, tumour necrosis factor α (TNF-α) has been attributed an active role in the pathogenesis of uveitis in several experimental animal models [2,3]. In the clinical setting, significantly higher levels of this proinflammatory cytokine have been determined in both serum (2.7-fold) and aqueous humour (AH) (25-fold) of patients with uveitis [1]. As TNF-α induces the expression of adhesion molecules, chemokines, and cytokines that are involved in the inflammatory process [4], its neutralization has been demonstrated to reduce macrophage infiltration, ultimately preventing tissue destruction in active uveitis [5].

Controlling ocular inflammation is therefore the goal of any treatment indicated for non-infectious uveitis (NIU), as persistent ocular inflammation can trigger severe visual impairments [6]. Currently, systemic corticosteroids are the first therapeutic option, followed by immunosuppressants, for NIU, but their long-term use puts patients at significant risk of adverse events. With the arrival of the first (actually, the only) anti-TNF-α biologic drug approved for the treatment of NIU in 2016, adalimumab marked an inflexion point in the clinical management of this inflammatory ocular disease [7]. Additionally, off-label use of infliximab in NIU has been demonstrated to control inflammation in up to 80% of patients with refractory uveitis with a good safety profile [8,9]. Golimumab has also demonstrated efficacy in anterior uveitis reducing the occurrence rate and disease activity [10] and non-infectious panuveitis in terms of the corticosteroid-sparing effect and reduced rate of relapses by 30% [11]. However, there are several factors that can compromise the response to systemic treatment with adalimumab in NIU patients: there is a limited and controversial relationship between systemic exposure to adalimumab and clinical outcomes [12,13], there are no therapeutic ranges defined for adalimumab in NIU [6], and there are immunogenicity issues with the corresponding production of antidrug–antibodies that do increase adalimumab systemic clearance [14] as well as the presence of blood–ocular barriers restricting ocular exposure to this class of biologic drugs [15]. Moreover, topical administration of biologic drugs does not provide significant intraocular drug concentrations either, as these macromolecules are not expected to penetrate the corneal epithelium and because of the rapid removal to the bloodstream by conjunctival blood and lymphatic vessels [16]. Consequently, intravitreal (IVT) injections are the only way to sort out the above restrictions, and the use of anti-TNF-α monoclonal antibodies (mAbs) deserves further investigation [4].

The ongoing phase 2 clinical trial “Intravitreal Adalimumab Versus Subcutaneous Adalimumab in Non-infectious Uveitis (IVAS)” (NCT02706704) has the primary objective of evaluating the efficacy of IVT adalimumab compared to subcutaneous (SC) adalimumab in patients with NIU. However, the IVT injection of mAbs in solution could be associated with many challenges such as high peak concentrations due to pulsed dosing, potential adverse events because of high concentrations of drug and the corresponding intensive dosing regimen requiring frequent administration (every 4 weeks), which is inherently associated with poor patient compliance and burden to the health care system [16]. Because sustained-release formulations can control the kinetics of the drug and minimize the amplitude between peaks and troughs derived from pulsed dosing, there is an urgent need for the development of suitable intraocular delivery systems for biologics in order to alleviate patient compliance and safety concerns [17].

Currently, as preclinical drug development strongly relies on in vivo animal experimentation [18], there is a global interest and an ongoing effort to develop novel methods aimed at replacing animal studies, reducing the number of animal studies to a minimum and refining the processes in order to minimize animal stress and suffering (also known as the 3Rs principles). In this sense, it is essential to develop predictive in vitro methods and simulation tools able to provide relevant data before in vivo testing. New approach methodologies (NAMs) refer to these novel methods (in vitro and in silico) that are compliant with the 3Rs principle of animal testing for the assessment of the efficacy and safety of new medicines [19]. Concisely, mechanistic models such as physiologically based pharmacokinetic (PBPK) models are fundamental for contextualizing NAMs outputs within clinical settings, thus allowing for a proper translation of model-based predictions to human outcomes [20]. Provided the well established use of PBPK models to answer “what if…?” questions throughout preclinical phases of drug development, the objective of the present work was to evaluate the kinetics of TNF-α in AH and iris-ciliary body (ICB) in silico and guide the design of sustained-release formulations of anti-TNF-α monoclonal antibodies (mAbs) for IVT administration in NIU diseased rats through a model-informed strategy.

## 2. Materials and Methods

**Figure 1:**
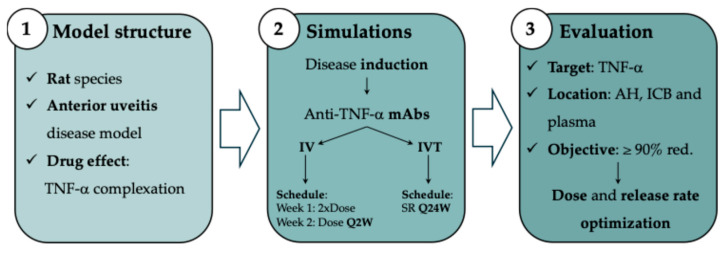
Modelling workflow for the design of sustained-release formulations of anti-TNF-α monoclonal antibodies for intravitreal administration in non-infectious uveitis. IV: intravenous; IVT: intravitreal; AH: aqueous humour; ICB: iris-ciliary body; and red: reduction.

A schematic diagram illustrating the model-informed preclinical design of sustained-release formulations of anti-TNF-α mAbs for IVT administration in NIU is depicted in Figure 1.

### 2.1. Model Structure

Using a previously developed endotoxin-induced (EIU) anterior uveitis disease model in rats [21], which is characterized by a time-dependent reduction in intraocular bulk flows (anterior-to-posterior cavities or $Q_{AV}$ and posterior-to-anterior cavities or $Q_{VA}$) due to iris inflammation as well as in AH drainage ($Q_{AH}$) because of ocular hypotony, a reversible higher permeability of blood–aqueous barrier as a consequence of the rupture of micro-vessels located at the ICB and a reversible systemic increase in TNF-α synthesis, we did upgrade the disease model removing the time dependencies in the TNF-α synthesis to generate a scenario characterized by a pathological condition instead of an induced and reversible systemic inflammation. TNF-α was considered as a soluble binding partner with zero-order synthesis ($k_{syn}$) and first-order degradation ($k_{deg}^{TNF}$) processes as shown below:

(1) $$\frac{dA_{TNF}}{dt}=k_{syn}^{NIU}-k_{deg}^{TNF}\cdot A_{TNF}(t)=SF\cdot k_{syn}^{Healthy}-k_{deg}^{TNF}\cdot A_{TNF}(t)$$

where $k_{syn}^{NIU}$ is the zero-order synthesis rate constant in NIU, $A_{TNF}(t)$ is the amount of free TNF-α at time t and SF is a synthesis factor that allows us to model the increased synthesis rate ($k_{syn}^{Healthy}$) because of systemic inflammation.

Since at steady-state TNF-α synthesis rate equals degradation rate,

$$k_{syn}^{Healthy}=k_{deg}^{TNF}\cdot A_{TNF}^{Healthy}\;so,$$

(2) $$\frac{dA_{TNF}}{dt}=k_{syn}^{NIU}-k_{deg}^{TNF}\cdot A_{TNF}(t)=SF\cdot k_{deg}^{TNF}\cdot A_{TNF}^{Healthy}-k_{deg}^{TNF}\cdot A_{TNF}(t)$$

where $A_{TNF}^{Healthy}$ represents the baseline levels of TNF-α in a healthy rat.

The physiological processes altered by the inflammatory status incorporated TNF-α complexation as pharmacodynamic (PD) effect in order to allow the system to recover its physiological condition following the equations:

(3) $$Q_{AH}\left(t\right)=\frac{Q_{AH}^{Healthy}}{a}+\left(Q_{AH}^{Healthy}-\frac{Q_{AH}^{Healthy}}{a}\right)\cdot \left(\frac{A_{complex}(t)}{{TNF}_{total}}\right)$$

(4) $$Q_{AV}\left(t\right)=\frac{Q_{AV}^{Healthy}}{b}+\left(Q_{AV}^{Healthy}-\frac{Q_{AV}^{Healthy}}{b}\right)\cdot \left(\frac{A_{complex}(t)}{{TNF}_{total}}\right)$$

(5) $$Q_{VA}\left(t\right)=\frac{Q_{VA}^{Healthy}}{c}+\left(Q_{VA}^{Healthy}-\frac{Q_{VA}^{Healthy}}{c}\right)\cdot \left(\frac{A_{complex}(t)}{{TNF}_{total}}\right)$$

(6) $$f_{Schlemm}\left(t\right)=f_{Schlemm}^{Disease}+\left(f_{Schlemm}^{Healthy}-f_{Schlemm}^{Disease}\right)\cdot \left(\frac{A_{complex}(t)}{{TNF}_{total}}\right)$$

(7) $$\sigma_{AH}\left(t\right)=\sigma_{AH}^{Disease}+\left(\sigma_{AH}^{Healthy}-\sigma_{AH}^{Disease}\right)\cdot \left(\frac{A_{complex}(t)}{{TNF}_{total}}\right)$$

where $Q_{AH}\left(t\right)$, $Q_{AV}\left(t\right)$, $Q_{VA}\left(t\right)$, $f_{Schlemm}\left(t\right)$, and $\sigma_{AH}\left(t\right)$ are the AH drainage, anterior-to-posterior intraocular flow, posterior-to-anterior intraocular flow, fraction of $Q_{AH}\left(t\right)$ through Schlemm’s canal and the reflection coefficient at the ICB’s vascular space/AH interface at time t, respectively. $Q_{AH}^{Healthy}$, $Q_{AV}^{Healthy}$, $Q_{VA}^{Healthy}$, $f_{Schlemm}^{Healthy}$ and $\sigma_{AH}^{Healthy}$ are the physiological values for AH drainage, anterior-to-posterior intraocular flow, posterior-to-anterior intraocular flow, fraction of $Q_{AH}^{Healthy}$ through Schlemm’s canal and the reflection coefficient at the ICB’s vascular space/AH interface, respectively. a, b and c are reduction coefficients to the physiological values of $Q_{AH}$, $Q_{AV}$ and $Q_{VA}$, respectively, because of the anterior uveitis. $f_{Schlemm}^{Disease}$ and $\sigma_{AH}^{Disease}$ are the fraction of $\frac{Q_{AH}^{Healthy}}{a}$ through Schlemm’s canal and reflection coefficient at the ICB vascular space/AH interface, respectively, in diseased rats. $A_{complex}(t)$ is the amount of drug–TNF-α complex formed in a 1:1 stoichiometry by means of a full target-mediated drug disposition model (TMDD):

(8) $$\frac{dA_{complex}}{dt}=\frac{k_{off}}{K_{d}}\cdot \frac{A_{TNF}(t)\cdot A_{drug}(t)}{V_{container}}-k_{off}\cdot A_{complex}(t)-k_{deg}^{complex}\cdot A_{complex}(t)$$

where $A_{complex}(t)$ is the amount of drug–TNF-α complex at time t, $k_{off}$ is the first-order dissociation rate constant of the complex, $K_{d}$ is the complex dissociation constant, $A_{drug}(t)$ is the amount of drug in the container of volume $V_{container}$ considered and $k_{deg}^{complex}$ is the first-order degradation rate constant of the drug–target complex. ${TNF}_{total}$ represents total TNF-α in the container, which is equal to the sum of free and complexed TNF-α:

(9) $${TNF}_{total}=A_{TNF}(t)+A_{complex}(t)$$

It is noteworthy that free TNF-α reduction as a consequence of the complexation by the biologic drug was considered at the anatomical location where the process is taking place, i.e., anterior chamber (AH) for $Q_{AH}$ and $f_{Schlemm}$, interstitial space of ICB for $Q_{AV}$ and $Q_{VA}$ and ICB plasma for $\sigma_{AH}$.

### 2.2. Model Parameters

Monoclonal antibodies (mAbs), TNF-α and mAbs-TNF-α complex model parameters are shown in Table 1. Provided the clinical benefit of TNF-α neutralization in NIU and the model performance of the adalimumab ocular model already developed [21], infliximab and golimumab were selected because they share the same structure (i.e., IgG1-related mAbs) and binding stoichiometry to TNF-α (i.e., 1:1) with adalimumab. Accordingly, the same first-order degradation rate constant was assumed for all drug–target complexes. Physiological and anatomical parameters’ values for rat species were directly taken from the Open Systems Pharmacology^®^ suite. Ocular anatomy and physiology were parameterized by allometrically scaling parameters’ values from rabbits as indicated in [21].

### 2.3. Simulations

Simulations consisted of the administration of the biologic drug in multiple dose regimens through intravenous (IV) or IVT injections to NIU diseased rats.

Firstly, a reference dose was established as the dose that caused free TNF-α to reduce by 90% at steady state in AH and ICB after IV administration in a multiple dose regimen. These simulations consisted of the administration of a loading dose (double the reference dose) after 1 week of disease induction followed by the administration of the reference dose every other week (Q2W) starting on week 2 over 1 year. This dosing regimen was selected as it is considered the standard of care (SOC) in the systemic treatment of NIU.

The IVT administration of the biologic drug was simulated as an IVT injection of a sustained-release formulation every 24 weeks (Q24W) over 1 year (~2 injections/year). To assess the effect of prolonging the ocular exposure to the biologic drug on free TNF-α, different zero-order release kinetics were evaluated in order to select a potential sustained-release formulation able to keep free TNF-α below 10% of pathological levels over 1 year in all ocular substructures affected in anterior uveitis. Once the optimal release rate was identified, and thus, the dose to be loaded in the sustained release formulation, the model was finally used to check the risk associated with possible formulation issues causing an increase in release rate, with the subsequent emptying of the formulation before the next administration. Inter-dose interval shortening that triggered the first TNF-α concentration above the therapeutic objective (i.e., 0.7 pM), all TNF-α concentrations above baseline (i.e., 1 pM) or all TNF-α levels reaching pathological concentrations (i.e., 7 pM) at the time of the next administration were determined. The perturbations of the posterior-to-anterior intraocular flow and AH drainage as a consequence of the temporal increase in intraocular pressure (IOP) triggered by the IVT administration were also considered as in the previous model [21].

The analysis was performed for all three anti-TNF-α mAbs (i.e., adalimumab, golimumab and infliximab) to check whether different binding kinetics to the target had a potential impact on TNF-α kinetics at the biophase (i.e., interstitial fluid of ICB). The modelling and simulation work was performed in MoBi^®^ 11 Update 3 (Open Systems Pharmacology^®^ suite). Deterministic simulations were run due to the lack of a proper description of interindividual variability of ocular anatomy and physiology in rat species.

### 2.4. Model Evaluation

Biologic drug exposure, TNF-α and drug–TNF-α complex kinetics were evaluated in the anterior chamber of the eye (AH), ICB (plasma and interstitial fluid) and venous plasma. Additionally, the average percentage reduction ($Red\left(\%\right)$) in free TNF-α from pathological levels at steady state was calculated in each of the abovementioned locations for both IV and IVT administrations as:

(10) $$Red\left(\%\right)=\frac{C_{TNF,avg}-C_{TNF}^{Disease}}{C_{TNF}^{Disease}}\cdot 100=\frac{{\left(C_{TNF}\right)}_{avg}-\left(SF\cdot C_{TNF}^{Healthy}\right)}{SF\cdot C_{TNF}^{Healthy}}\cdot 100$$

where $C_{TNF,avg}$ is the average concentration of TNF-α at steady state, $C_{TNF}^{Disease}$ refers to the pathological concentration of TNF-α as a consequence of an accelerated synthesis because of systemic inflammation (SF = 7) and $C_{TNF}^{Healthy}$ being the baseline concentration of TNF-α in a healthy individual. A therapeutic objective of a minimum 90% reduction in free TNF-α from pathological levels was established to guide dosing regimen design. Additionally, free TNF-α over free drug concentration ratio in each location was also evaluated for both routes of administration (ROA).

Data analyses conducted to perform graphical and numerical evaluation of simulations were done in RStudio (R version 4.5.1).

## 3. Results

**Figure 2:**
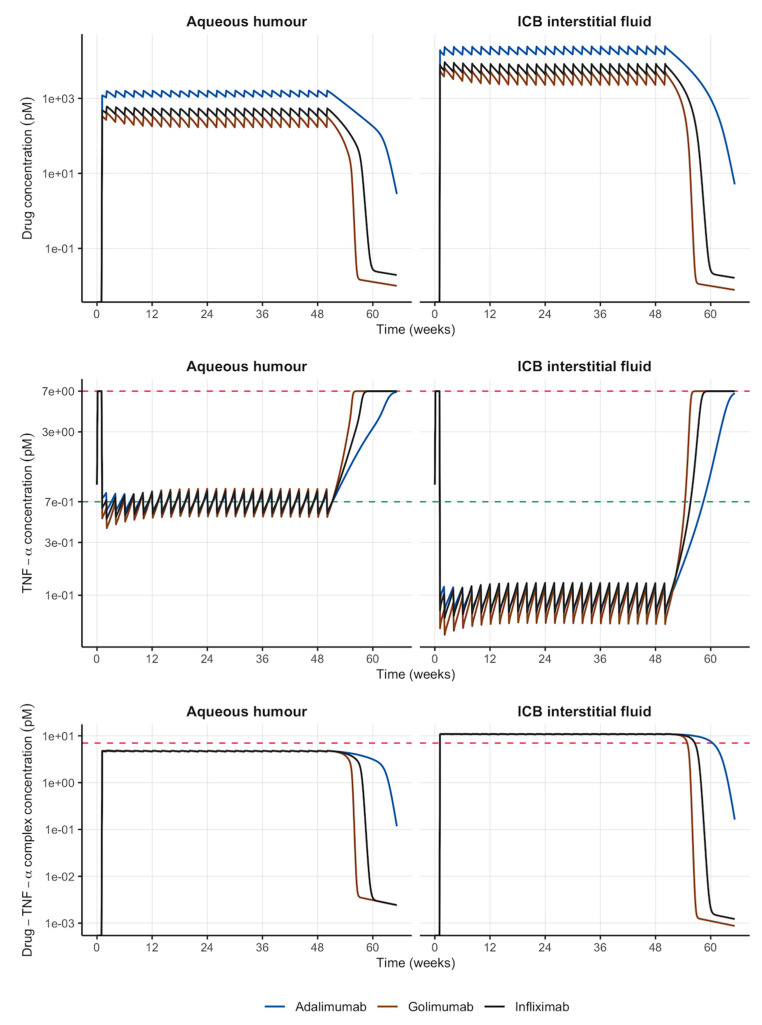
Simulated longitudinal profiles of drug, TNF-α and drug–TNF-α complex in the anterior chamber (aqueous humour) and ICB interstitial fluid over 1 year after the IV administration of reference doses of different anti-TNF-α mAbs. Red horizontal dashed line: TNF-α pathological concentration (7 pM); green horizontal dashed line: treatment goal (10% of TNF-α pathological concentration). ICB: iris-ciliary body; IV: intravenous; and mAbs: monoclonal antibodies.

The simulation of the IV administration of a loading dose (double the maintenance dose) on week 1 followed by maintenance doses Q2W starting on week 2 predicted a rapid establishment of steady-state drug concentration in AH and ICB interstitial fluid (Figure 2 top). However, the average drug concentration at steady-state in the anterior chamber (AH) of the eye was predicted to be significantly lower (14-fold) than the exposure in ICB interstitial fluid for all three mAbs (Table 2). Once the system achieved pathological levels of TNF-α (i.e., 7 pM) on week 1, these intensive dosing schedules were able to keep free TNF-α concentration below 10% of pathological values (i.e., 0.7 pM) in the AH and the biophase (i.e., interstitial fluid of ICB) over 1 year (Figure 2 middle). Moreover, the graphical evaluation of the longitudinal profiles of the drug–target complexes at the biophase revealed not only a persistent degree of TNF-α complexation with negligible variations in complex concentrations but also steady-state concentrations in ICB higher than TNF-α pathological levels, thus anticipating TNF-α is complexed as it is just synthesized (Figure 2 bottom). According to the predefined criteria, these dose levels (i.e., 24.5 μg for adalimumab, 7.4 μg for golimumab and 10.1 μg for infliximab) were selected as the reference doses for the systemic treatment of NIU diseased rats (Table 3).

**Figure 3:**
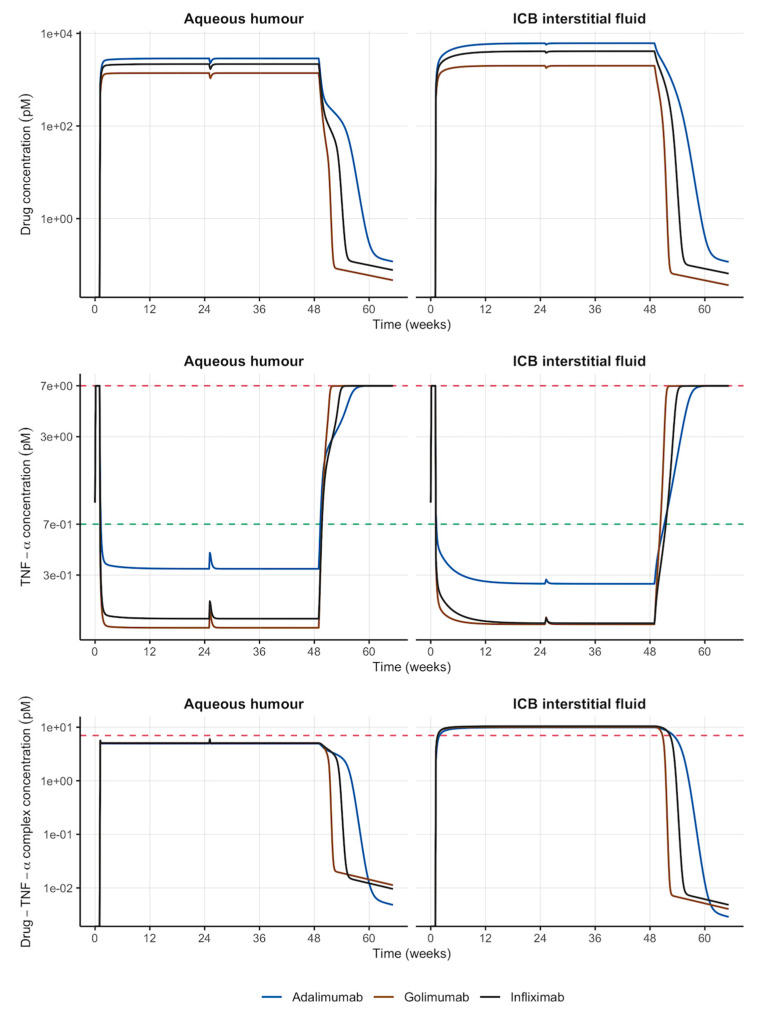
Simulated longitudinal profiles of drug, TNF-α and drug–TNF-α complex in the anterior chamber (aqueous humour) and ICB interstitial fluid over 1 year after the IVT administration of sustained-release formulations of different anti-TNF-α mAbs. Red horizontal dashed line: TNF-α pathological concentration (7 pM); green horizontal dashed line: treatment goal (10% of TNF-α pathological concentration). ICB: iris-ciliary body; IVT: intravitreal; mAbs: monoclonal antibodies.

The simulation of the IVT administration Q24W of sustained-release formulations containing any of the mAbs evaluated predicted a higher exposure in the anterior chamber (AH) of the eye, with 2.13-, 5.79- and 5.04-fold higher steady-state drug concentrations for adalimumab, golimumab and infliximab, respectively, when compared to the IV administration (Table 2). Moreover, the simulation of the IVT administration predicted the highest exposure in interstitial fluid of ICB (i.e., the biophase), with a significant reduction in systemic plasma exposure for adalimumab (79%), golimumab (74%) and infliximab (60%) (see Table 2). Sustained-release formulations directly injected into the vitreous releasing adalimumab, golimumab or infliximab at 1.802, 0.979 or 1.442 μg/week, respectively, were also able to keep TNF-α below 10% of the pathological levels at the biophase (Figure 3 middle). Even though TNF-α reduction in plasma of ICB was not as pronounced as after IV administration, it still predicted a >90% reduction in TNF-α from pathological levels (Table 3). Individual plots for each drug after IV or IVT administration including venous and ICB plasma are shown in Supplementary Material Figures S1 and S2.

**Figure 4:**
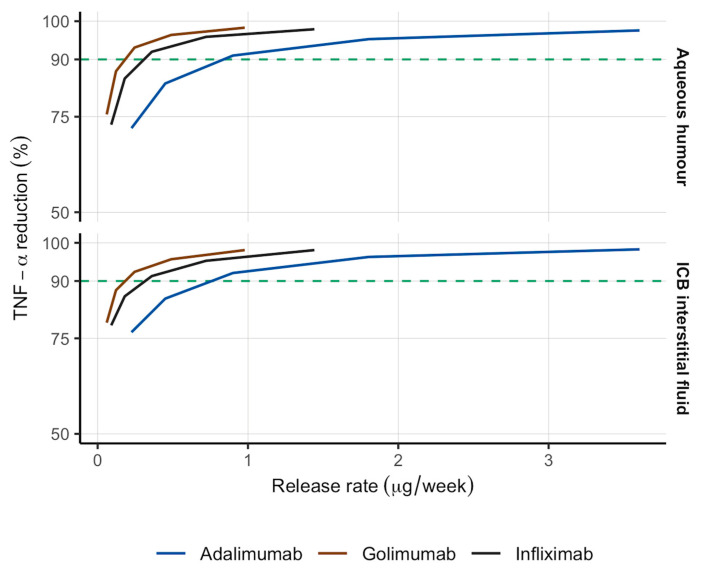
Predicted TNF-α percent reductions in the anterior chamber (aqueous humour) and ICB interstitial fluid over 1 year after the IVT administration of different sustained-release formulations of adalimumab, golimumab and infliximab. Green horizontal dashed line: treatment goal (90% TNF-α reduction). ICB: iris-ciliary body.

As shown in Table 3, half of these release rates (i.e., 0.900, 0.489 and 0.721 μg/week for adalimumab, golimumab and infliximab, respectively) were also anticipated to attain the therapeutic goal in the AH as well as in the interstitial fluid of ICB, but the average reduction in TNF-α in plasma of ICB resulted in 84.4, 83.3% and 87.6 for adalimumab, golimumab and infliximab, respectively. Lower release rates did not meet the goal of more than 90% reduction in free TNF-α in any of the ocular structures affected in anterior NIU (Figure 4). Accordingly, release rates higher than 0.900, 0.489 and 0.721 μg/week for adalimumab, golimumab and infliximab, respectively, could be selected as potential release rates for sustained-release formulations for IVT injection in NIU because they entail significantly lower total dose over 1 year of treatment, with percent reductions as high as 93.5 for adalimumab, 88.2 for golimumab and 87.3 for infliximab (see Table 3). A local sensitivity analysis on parameters driving disease status (i.e., SF) and drug effect (i.e., k_off_ and K_D_) through 2-, 4-, and 10-fold changes and their impact on intravitreal release rate optimization is reported in the Supplementary Material. The results reveal SF as the parameter IVT sustained-release formulations’ release rate is most sensitive to, since higher release rates are needed in order to properly attain the therapeutic objective previously defined. Regarding the mAbs, infliximab has been identified as the biologic drug whose release rate is less sensitive to uncertainty in these parameters, while the adalimumab release rate has shown the highest sensitivity. Notwithstanding, this sensitivity analysis shows high robustness of model outcomes to uncertainty in the parameters mainly influencing pharmacodynamic response.

**Figure 5:**
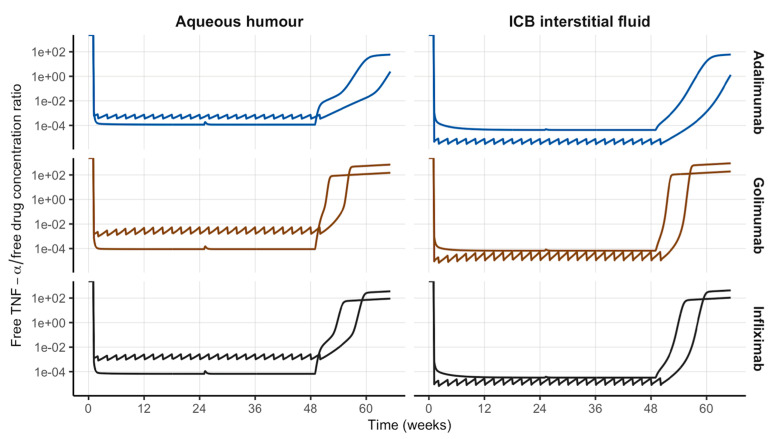
Simulated longitudinal profiles of free TNF-α over free drug concentration ratio in the anterior chamber (aqueous humour) and ICB interstitial fluid after the IV administration of reference doses following SOC dosing schedule (sharp profiles) or IVT administration of sustained-release formulations Q24W (smooth profiles) of different monoclonal antibodies. ICB: iris-ciliary body; SOC: standard of care; Q24W: every 24 weeks.

Moreover, the longitudinal profiles of free TNF-α over free drug concentration ratios in both the anterior chamber (AH) and ICB reveal IVT administration of sustained-release formulations can significantly reduce free TNF-α over 1 year as the more intensive IV dosing regimen, with a negligible perturbation at week 24 post-dose when the second injection is administered (Figure 5). Ratios in systemic and ICB plasma are shown in Supplementary Material Figure S3.

**Figure 6:**
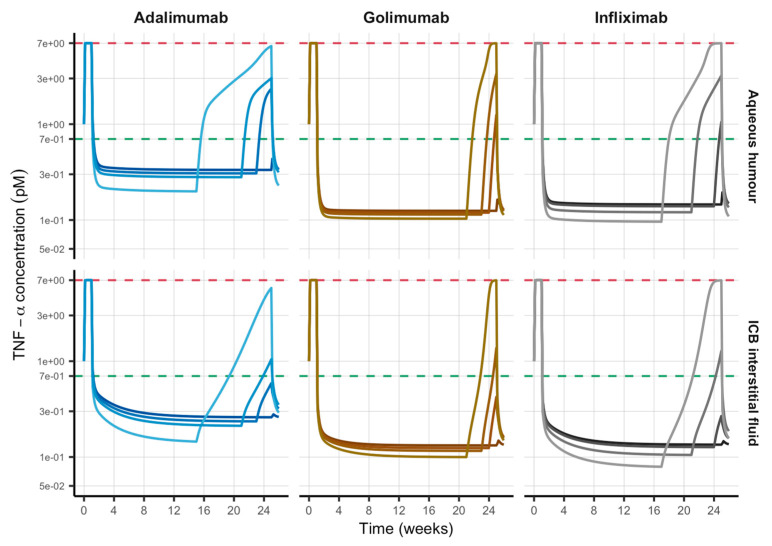
Impact of release rate of anti-TNF-α monoclonal antibodies on TNF-α kinetics in aqueous humour and ICB interstitial fluid after the IVT administration of sustained-release formulations. Dark profiles correspond to the optimal release rate selected; lighter profiles relate to different degrees of release rate increases (see Table 4). IVT: intravitreal; ICB: iris-ciliary body.

Graphical evaluation of TNF-α kinetics in AH and interstitial fluid of ICB at different times of formulation depletion before next IVT administration is shown in Figure 6. Systemic and ICB plasma longitudinal profiles are shown in Supplementary Material Figure S4. As can be seen, the higher the release rate the higher the TNF-α reduction from pathological levels regardless of the mAb evaluated. However, as the release rate increases from previously selected optimal values (darkest profiles), the formulation empties before next administration (i.e., 24 weeks post-dose) and triggers free TNF-α concentration not only to be over the therapeutic objective but also reaching pathological levels for extremely rapid release rates (lightest profiles). Table 4 summarizes different TNF-α-related outcomes at the time of next administration triggered by potential formulation issues causing an increase in release rate. TNF-α levels in the anterior chamber were the first being affected when formulation had run out of drug as early as 23 weeks post-dose for adalimumab, golimumab and infliximab, thus causing TNF-α concentration in AH to be above the therapeutic objective (i.e., 0.7 pM) at time of next administration (1.66, 1.25 and 1.05 pM, respectively) (see Figure 6). In order to reach TNF-α concentrations in AH, ICB (plasma and interstitial fluid) and venous blood above baseline at the time of next administration, IVT sustained-release formulations releasing adalimumab, golimumab or infliximab would have to be empty of drug at week 20, 22 and 20, respectively. Finally, release duration shortening as high as 10, 4 and 8 weeks for adalimumab, golimumab and infliximab with relative release rate increases of 71, 20 and 50%, respectively, was anticipated to cause TNF-α concentration reaching pathological levels at the time of next administration (i.e., 24 weeks post-dose).

## 4. Discussion

In this work, a bottom-up approach using a PBPK-PD model for different anti-TNF-α mAbs has been performed in order to in silico assess the impact of the release rate of sustained-release formulations for IVT administration on the kinetics of TNF-α in the anterior chamber of the eye and ICB of NIU diseased rats. The PBPK-PD model incorporates a detailed description of the anatomy and physiology of the ocular tissue as reported by Bussing and Shah [25]. Apart from accounting for the perturbation in vitreous humour volume, intraocular flows and AH drainage through the Schlemm’s canal triggered by the IVT administration, the disease model accounts for an increased synthesis rate of TNF-α because of a systemic inflammation, the reduction in posterior-to-anterior and anterior-to-posterior intraocular flows as a direct consequence of iritis, the disruption of the blood–ocular barrier located at the ICB, and the ocular hypotony in early stages of the disease [21]. Regarding the TMDD model, we considered TNF-α as a stationary molecule assuming there is no distribution between the different tissues’ substructures. Because of this, the TMDD model did not include additional processes such as endosomal uptake/recycle, FcRn binding and trans-capillary transport through small and large pores of the endothelial wall of TNF-α. Notwithstanding, this kind of static TMDD models have been successfully applied to describe the pharmacokinetics of tau protein-directed mAbs in plasma, cerebrospinal fluid and brain interstitial fluid of rats, monkeys and humans with high accuracy after the administration of different dose levels, being also able to predict the target occupancy achieved at the biophase [26].

The strategy here reported follows the alternative preclinical development workflow proposed by Mehta et al. [18] by which “a priori in silico” approach integrating standard and advanced in vitro experiments to inform computational models that incorporate drug-, physiological- and disease-specific parameters derived from prior data, models and knowledge can be used to perform in vivo predictions that guide the design of optimal preclinical studies, including the determination of dose, dosing regimens, biomarkers, endpoints and sample times. With this level of detail of the mechanisms and processes driving the ocular PK and PD of the biological drug in diseased animals, this PBPK-PD model enables the assessment of drug exposure at the biophase (i.e., interstitial fluid of ICB) not only in preclinical stages of drug development to support proof-of-concept results, but also early in drug discovery where new modalities or alternative drugs with different target binding kinetics could be efficiently tested, identified and proposed to move forward in the drug development process.

Currently, the clinical management of adult patients with NIU with anti-TNF-α mAbs consists of SC injections of adalimumab at an initial dose of 80 mg followed by 40 mg every other week starting one week after the loading dose (SOC regimen). Infliximab and golimumab are only approved for the treatment of inflammatory disorders like rheumatoid arthritis, ulcerative colitis, ankylosing spondylitis or psoriatic arthritis after IV (infliximab) or SC (golimumab) administrations, with no indication for NIU. However, they were selected because both drugs share molecular structure (IgG1-related) and target (TNF-α) with adalimumab and pretended to anticipate TNF-α kinetics at the biophase with alternative (but already developed) anti-TNF-α mAbs. As both adalimumab and golimumab exhibit moderate absolute bioavailability after SC administration (64 and 51%, respectively) (EMA SmPC Humira^®^ and Gobivaz^®^), the IV administration was selected as the reference ROA to which to compare IVT injections in order to avoid any possible difference in ocular exposure due to partial absorption. As the allometric scaling of dose is not recommended for biological drugs that exhibit significant target-binding effects [27], a theoretical reference dose was selected after simulating the IV administration of different multiple-dose regimens of the mAbs evaluated. Once the disease status was reached at week 1, an IV dose of 24.5, 7.4 or 10.1 μg of adalimumab, golimumab or infliximab, respectively, administered every 2 weeks after a loading double dose was able to keep free TNF-α concentration in the anterior chamber of the eye and ICB below 90% of pathological levels over 1 year (Figure 2). However, this dosing regimen required 26 injections per year to maintain the therapeutic goal, which would compromise patients’ adherence and ultimately, clinical response. Alternatively, IVT injections were evaluated through model-based simulations of the administration of sustained-release formulations in order to overcome anterior clearance of mAbs and prolong ocular exposure to the anti-TNF-α biological drug.

Controlling drug release is a central goal in IVT drug delivery, as long dosing intervals are desired in order to minimize the burden of injections to the patients and the health care system [28]. Pharmacokinetic simulations can be used in the design of drug delivery systems for IVT administration, as once the ocular clearance is known it is then possible to simulate different release rates or drug loading in the delivery system to anticipate the ocular exposure achieved in each scenario [15]. Moreover, a sufficiently detailed ocular model accounting for the different anatomical locations and physiological processes that characterize this complex organ and coupled with a physiological representation of the organism (i.e., a PBPK model), allows for making predictions not only in sampling fluids like aqueous or vitreous humours but also at very challenging sites like ICB plasma or interstitial fluid. The flexibility of these models to simulate different drugs in the same species, their unique performance in inter-species translation, their ability to incorporate disease-related physiological changes and the corresponding impact on PK of drugs together with the growing amount of in vitro and in silico data needed, could provide significant advantages in the field of IVT drug administration [15]. As can be seen in Figure 3 and Table 2, the IVT administration of sustained-release formulations allows us to achieve significantly higher exposure to the biological drugs in the anterior chamber of the eye (AH), consequently leading to a higher degree of TNF-α complexation and reduction. Since all three mAbs evaluated share the same chemical structure (i.e., IgG1-related), size (i.e., 144–146 KDa) and main elimination pathway (i.e., anterior clearance), the different release rates needed in order to attain the therapeutic objective of 90% reduction of TNF-α from pathological levels are likely due to its binding affinity (k_on_) to the target calculated as k_off_/K_D_. Accordingly, the following rank of release rates can be defined with the higher the release rate, the lower the k_on_: adalimumab > infliximab > golimumab.

When evaluating ICB interstitial fluid longitudinal profiles, simulations predicted a higher exposure to the mAbs after the IV administration but with a negligible impact on TNF-α complexation, thus not impacting therapeutic goal achievement at the biophase (see Table 2). Moreover, systemic exposure after the IVT administration is significantly reduced, with steady-state concentrations significantly lower than after the IV administration, thus minimizing the risk of possible off-target adverse effects. At this point, it has to be noted that IVT administration of the proposed sustained-release formulations containing adalimumab, golimumab and infliximab resulted in a total dose reduction of 93.5%, 88.1% and 87.3% per year, respectively, and an obvious reduction in the number of administrations, thus anticipating higher patience compliance and the need of lower resources for the health care system.

Predictions of TNF-α percent reductions in AH and interstitial fluid of ICB were very close after IVT administration of the drugs evaluated (see Figure 3 middle and Table 3) because of the easy access to the anterior chamber from the posterior cavity of the eyeball (administration site). However, when the mAbs were administered intravenously, the model predicted a 10% difference between TNF-α reduction in the anterior chamber (AH) and interstitial fluid of ICB most likely because of a direct consequence of the selective permeability of the blood–aqueous barrier that limits the mAb mass transfer from the bloodstream to the AH. This correspondence is confirmed by the similar behaviour of the free TNF-α over free mAb concentration ratio at steady state in AH and interstitial fluid of ICB (Figure 5). This finding would anticipate TNF-α concentration in AH as a possible surrogate marker of TNF-α levels at the biophase in anterior NIU (i.e., ICB interstitial fluid) when the anti-TNF-α mAb is administered by IVT injection, making possible dose adjustments as per indirect determination of drug concentration in the anterior chamber of the eye (AH). Furthermore, our model-based evaluation of formulation issues that can lead to unexpected escalations in the release rate of the loaded dose, resulting in premature emptying prior to scheduled administrations, or challenges such as system obstruction due to biofouling or antibody degradation, which can precipitate the premature cessation of release, has elucidated the anterior chamber of the eye as the most sensitive anatomical location to formulation issues. This is evidenced by the observation that TNF-α concentration in AH was the primary outcome to exceed the therapeutic objective for all three mAbs evaluated with release rate increases as low as 4%.

Among the drugs evaluated, golimumab was predicted to be the most sensitive to formulation issues, since TNF-α in AH, ICB (plasma and interstitial fluid) and venous blood were predicted to be above baseline after the administration of formulations with an increase in release rate of only 9% (compared to the 20% increase needed by adalimumab and infliximab), and anticipated reaching pathological levels when drug release was interrupted 4 weeks before the next administration. These results can be attributed to its binding kinetics to TNF-α, as golimumab binds TNF-α the fastest (lowest k_on_ calculated as k_off_/K_D_, i.e., 0.0186 h^−1^ pM^−1^) and forms the strongest drug–TNF-α complex (lowest K_D_, i.e., 18 pM), meaning an advantage in terms of the dose required to achieve the therapeutic objective (lowest dose, see Table 3) but having the drawback of being more rapidly eliminated through specific and target-mediated pathways when compared to infliximab or adalimumab (higher doses loaded to the formulation, see Table 3). So, an early assessment of changes in release rate for sustained-release formulations of anti-TNF-α mAbs for IVT administration is advisable in order to optimize the dose loaded and balance therapeutic objective attainment and possible emptying of formulation before the next injection.

Different delivery systems of biologic drugs into the eye do exist (e.g., biodegradable polymers and implants, reservoir systems and gels, microspheres, devices and non-erodible implants) and good reviews discussing their advantages, drawbacks and current development status can be found elsewhere [16,17,29]. Among them, intraocular devices such as Port Delivery Systems represent an innovative refillable ocular implant that allows for the control of the release rate of, for example, ranibizumab and have been approved by the US Food and Drug Administration for the treatment of neovascular age-related macular degeneration in adults, since refill-exchanges Q24W were equivalent and non-inferior to flat doses IVT administered Q4W [30]. These devices do not require any modification of the drug or complex formulation technology, thus allowing the use of previously developed biologic drugs [16], as it could be the case for the mAbs here evaluated, and expanding the treatment landscape of ocular inflammatory disorders.

However, this PBPK-PD model has several limitations that must be highlighted. Our model assumes a stable disease progression with a constant synthesis factor that does not consider time-varying effects on the underlying inflammatory disease. On the other hand, the current PBPK-PD framework does not allow for predicting uveitis flares due to the development of tolerance/resistance mechanisms. Notwithstanding, this framework is flexible enough to incorporate these processes once experimental information becomes available on how tolerance or anti-drug antibodies could compromise disease control by free TNF-α complexation. In this regard, further validation of the disease model and more experimental evidence regarding possible safety concerns associated with sustained intravitreal exposure to adalimumab, golimumab and infliximab, including immunogenicity or ocular toxicity, as well as its long-term stability in intraocular delivery systems is needed in order to efficiently translate these results to the clinical setting.

## 5. Conclusions

The modelling strategy here reported follows the principles of new approach methodologies in preclinical drug development and could be used to conduct well designed and more efficient in vivo studies aimed to assess the efficacy of sustained-release formulations for intravitreal administration in the treatment of ocular inflammatory disorders, with less animal testing and without compromising the accuracy of the model-informed predictions for human translation. Notwithstanding, this strategy would require experimental data in order to validate the disease model and to assess possible safety and drug stability concerns due to their long-term use in intraocular delivery systems to efficiently translate these results to the clinical setting.

## Figures and Tables

**Table 1:** Drug, target and drug–target complex parameters for the PBPK-PD model.

Parameter (Units)	Adalimumab [Reference/Comments]	Golimumab [Reference/Comments]	Infliximab [Reference/Comments]
MW (KDa)	144.19 [DrugBank]	146.94 [DrugBank]	144.19 [DrugBank]
Radius (nm)	5.04 [Predicted from MW]	5.08 [Predicted from MW]	5.04 [Predicted from MW]
Structure	IgG1-related mAb	IgG1-related mAb	IgG1-related mAb
K_D_ FcRn (μM)	1.18 [22]	0.63 [23]	0.80 [22]
k_ass_ FcRn (l/μmol/min)	0.87 [Default]	0.87 [Default]	0.87 [Default]
k_uptake_ (1/min)	0.29 [Default]	0.29 [Default]	0.29 [Default]
k_recycling_ (1/min)	0.09 [Default]	0.09 [Default]	0.09 [Default]
K_D_ TNF-α (pM)	127 [24]	18 [24]	44 [24]
k_off_ TNF-α (1/h)	0.576 [24]	0.335 [24]	0.720 [24]
TNF-α			
MW (kDa)	52 [Novusbio]	52 [Novusbio]	52 [Novusbio]
Baseline concentration (pM)	1 [21]	1 [21]	1 [21]
k_deg_ (1/h)	0.223 [23]	0.223 [23]	0.223 [23]
SF	7 [21]	7 [21]	7 [21]
Drug–TNF-α complex			
MW (kDa)	196.19 [Calculated]	198.94 [Calculated]	196.19 [Calculated]
Radius (nm)	5.74 [Predicted from MW]	5.78 [Predicted from MW]	5.74 [Predicted from MW]
k_deg_ (1/h)	0.18 [23]	0.18 [23]	0.18 [23]

MW: molecular weight; K_D_ FcRn: FcRn dissociation constant; k_ass_: second-order association rate constant to FcRn; k_uptake_: first-order uptake rate constant to endosomes from vascular and interstitial spaces; k_recycling_: first-order recycling rate constant from endosomes to vascular and interstitial spaces; K_D_ TNF-α: TNF-α dissociation constant; k_off_ TNF-α: first-order dissociation rate constant from TNF-α; k_deg_ target: first-order degradation rate constant of TNF-α; SF: synthesis factor; and k_deg_ complex: first-order degradation rate constant of drug–target complex.

**Table 2:** Predicted steady-state drug concentrations in different ocular substructures and systemic plasma after the IV or IVT administration of adalimumab, golimumab or infliximab.

Drug	Adalimumab				Golimumab				Infliximab			
Location	AH	ICB_int_	ICB_plasma_	Venous Plasma	AH	ICB_int_	ICB_plasma_	Venous Plasma	AH	ICB_int_	ICB_plasma_	Venous Plasma
$C_{avg,ss}^{IV}$ (pM)	1350	18,880	19,690	19,690	240	3470	3630	3630	430	6050	6320	6320
$C_{ss}^{IVT}$ (pM)	2870	6100	4150	4150	1390	1990	930	930	2170	4100	2550	2550
Fold change	2.13	0.32	0.21	0.21	5.79	0.57	0.26	0.26	5.04	0.68	0.40	0.40

AH: aqueous humour; ICB_int_: iris-ciliary body interstitial fluid; ICB_plasma_: iris-ciliary body plasma; IV: intravenous; $C_{avg,ss}^{IV}$: average drug concentration at steady state after IV administration of reference doses Q2W; IVT: intravitreal; $C_{ss}^{IVT}$: drug concentration at steady state after the IVT administration of the sustained-release formulations selected. Fold change: $C_{ss}^{IVT}$/$C_{avg,ss}^{IV}$.

**Table 3:** TNF-α percent reductions in the anterior chamber (AH) of the eye and ICB after the IV or IVT administration of different formulations of adalimumab, golimumab and infliximab following different dosing schedules.

Drug	ROA	Dosing Regimen	N_adm_	Dose (μg)	Release Rate (μg/Week)	Total Dose (μg)	Average TNF-α Reduction (%)			
							AH	ICB_int_	ICB_plasma_	Venous Plasma
**Adalimumab**	**IV**	**SOC**	**26**	**24.5**	**-**	**661.8**	**90.3**	**98.7**	**98.5**	**98.5**
Adalimumab	IVT	Q24W	2	-	0.225	10.8	72	76.6	43.1	43.1
Adalimumab	IVT	Q24W	2	-	0.450	21.6	83.7	85.4	66.6	66.6
Adalimumab	IVT	Q24W	2	-	0.900	43.3	91	92.1	84.4	84.4
Adalimumab	IVT	Q24W	2	-	1.802	86.5	95.3	96.3	93.7	93.7
Adalimumab	IVT	Q24W	2	-	3.605	147.1	97.6	98.3	97.3	97.3
**Golimumab**	**IV**	**SOC**	**26**	**7.4**	**-**	**198.4**	**90.1**	**98.8**	**98.7**	**98.7**
Golimumab	IVT	Q24W	2	-	0.060	2.9	75.6	79.1	16.6	16.6
Golimumab	IVT	Q24W	2	-	0.122	5.9	86.9	87.6	33.4	33.4
Golimumab	IVT	Q24W	2	-	0.245	11.8	93.1	92.4	59.6	59.6
Golimumab	IVT	Q24W	2	-	0.489	23.5	96.4	95.7	83.3	83.3
Golimumab	IVT	Q24W	2	-	0.979	47.0	98.3	98.1	95.4	95.4
**Infliximab**	**IV**	**SOC**	**26**	**10.1**	**-**	**272.5**	**90.1**	**98.6**	**98.4**	**98.4**
Infliximab	IVT	Q24W	2	-	0.090	4.3	72.9	78.4	22.4	22.4
Infliximab	IVT	Q24W	2	-	0.180	8.7	85	86	42.9	42.9
Infliximab	IVT	Q24W	2	-	0.360	17.3	92	91.3	68.4	68.4
Infliximab	IVT	Q24W	2	-	0.721	34.6	95.9	95.3	87.6	87.6
Infliximab	IVT	Q24W	2	-	1.442	69.2	97.9	98.1	96.4	96.4

ROA: route of administration; N_adm_: number of administrations over 1 year; AH: aqueous humour; ICB_int_: iris-ciliary body interstitial fluid; ICB_plasma_: iris-ciliary body plasma; Bold text: reference doses; IV: intravenous; SOC: standard of care; IVT: intravitreal; and Q24W: every 24 weeks.

**Table 4:** Number of weeks without drug release before the next administration triggering different TNF-α outcomes.

TNF-α Outcome at Next Administration (Week 24 Post Dose)	All Levels Below Therapeutic Objective (Weeks; Release Rate (μg/Week))	First Level Above Therapeutic Objective (Weeks; Release Rate (μg/Week)) (Location)	All Levels Above Baseline Concentration (Weeks; Release Rate (μg/Week))	All Levels at Pathological Concentration (Weeks; Release Rate (μg/Week))
Adalimumab	0; 1.802	1; 1.880 (AH)	4; 2.163	10; 3.089
Golimumab	0; 0.979	1; 1.022 (AH)	2; 1.068	4; 1.175
Infliximab	0; 1.442	1; 1.504 (AH)	4; 1.730	8; 2.163

AH: aqueous humour.

## Data Availability

The original contributions presented in this study are included in the article/Supplementary Materials. Further inquiries can be directed to the corresponding author.

## Supplementary Materials

The following supporting information can be downloaded at: https://www.mdpi.com/article/10.3390/pharmaceutics18040445/s1. Figure S1. Simulated longitudinal profiles of drug, TNF-α and, drug-TNF-α complex in the anterior chamber (aqueous humour), ICB (interstitial fluid and plasma) and venous plasma over 1 year after the IV administration of reference doses of different anti-TNF-α mAbs; Figure S2. Simulated longitudinal profiles of drug, TNF-α and, drug-TNF-α complex in the anterior chamber (aqueous humour), ICB (interstitial fluid and plasma) and venous plasma over 1 year after the IVT administration of sustained release formulations of different anti-TNF-α mAbs; Figure S3. Simulated longitudinal profiles of free TNF-α over free drug concentration ratio in the anterior chamber (aqueous humour), ICB (interstitial fluid and plasma) and venous plasma after the IV administration of reference doses following SOC dosing schedule (sharped profiles) or IVT administration of sustained release formulations Q24W (smooth profiles) of different monoclonal antibodies; Figure S4. Impact of release rate of anti-TNF-α monoclonal antibodies on TNF-α kinetics in the anterior chamber (aqueous humour), ICB (interstitial fluid and plasma) and venous plasma after the IVT administration of sustained release formulations.

## Abbreviations

The following abbreviations are used in this manuscript:

3Rs	Replace, reduce, refine
AH	Aqueous humour
EIU	Endotoxin-induced uveitis
ICB	Iris-ciliary body
IgG1	Immunoglobulin G1
IOP	Intraocular pressure
IV	Intravenous
IVT	Intravitreal
mAb	Monoclonal antibody
NAMs	New approach methodologies
NIU	Non-infectious uveitis
PBPK	Physiologically based pharmacokinetics
PD	Pharmacodynamics
PK	Pharmacokinetics
Q24W	Every 24 weeks
Q2W	Every 2 weeks
ROA	Route of administration
SC	Sub-cutaneous
SOC	Standard of care
TMDD	Target-mediated drug disposition
TNF-α	Tumour necrosis factor a
