# Supplementary material for: Model-Based Design of Sustained-Release Formulations of Anti-TNF-α Monoclonal Antibodies for Intravitreal Administration
Source: Pharmaceutics. 2026 Apr 4;18(4):445. doi: 10.3390/pharmaceutics18040445 (PMC13118353; doi:10.3390/pharmaceutics18040445)
Supplement: Supplementary file 1 [file pharmaceutics-18-00445-s001.zip › pharmaceutics-4190239-supplementary.pdf]

Model-Based Design of Sustained-Release Formulations of Anti-TNF-α Monoclonal Antibodies for Intravitreal Administration

Javier Reig-López, Marina Cuquerella-Gilabert, Javier Zarzoso-Foj, Víctor Mangas-Sanjuán, Virginia Merino and Matilde Merino-Sanjuán

Supplementary Material

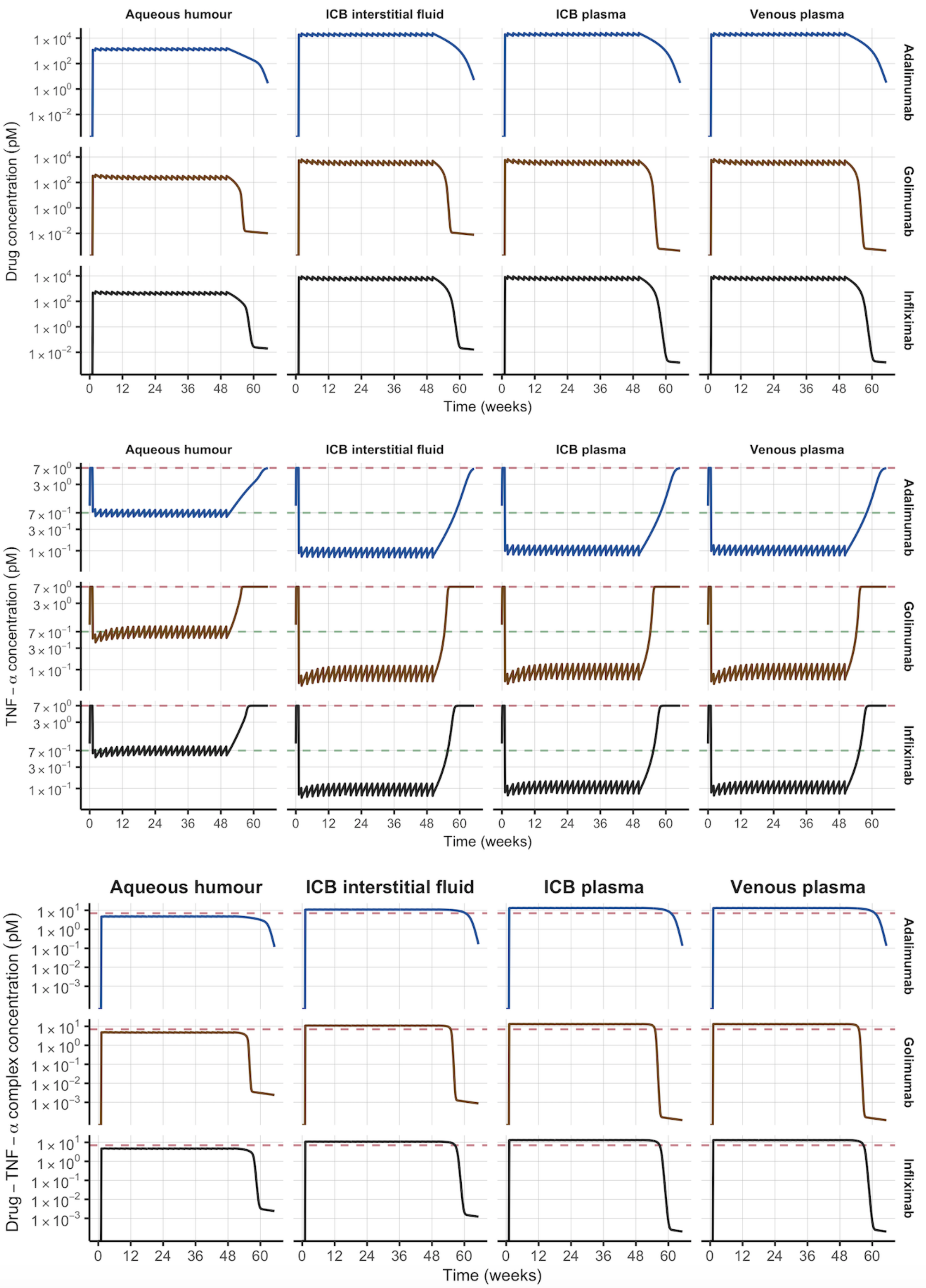

Figure S1. Simulated longitudinal profiles of drug, TNF-α and, drug-TNF-α complex in the anterior chamber (aqueous humour), ICB (interstitial fluid and plasma) and venous plasma over 1 year after the IV administration of reference doses of different anti-TNF-α mAbs. Red horizontal dashed line: TNF-α pathological concentration (7 pM); green horizontal dashed line: treatment goal (10% of TNF-α pathological concentration). ICB: iris-ciliary body; IV: intravenous; mAbs: monoclonal antibodies.

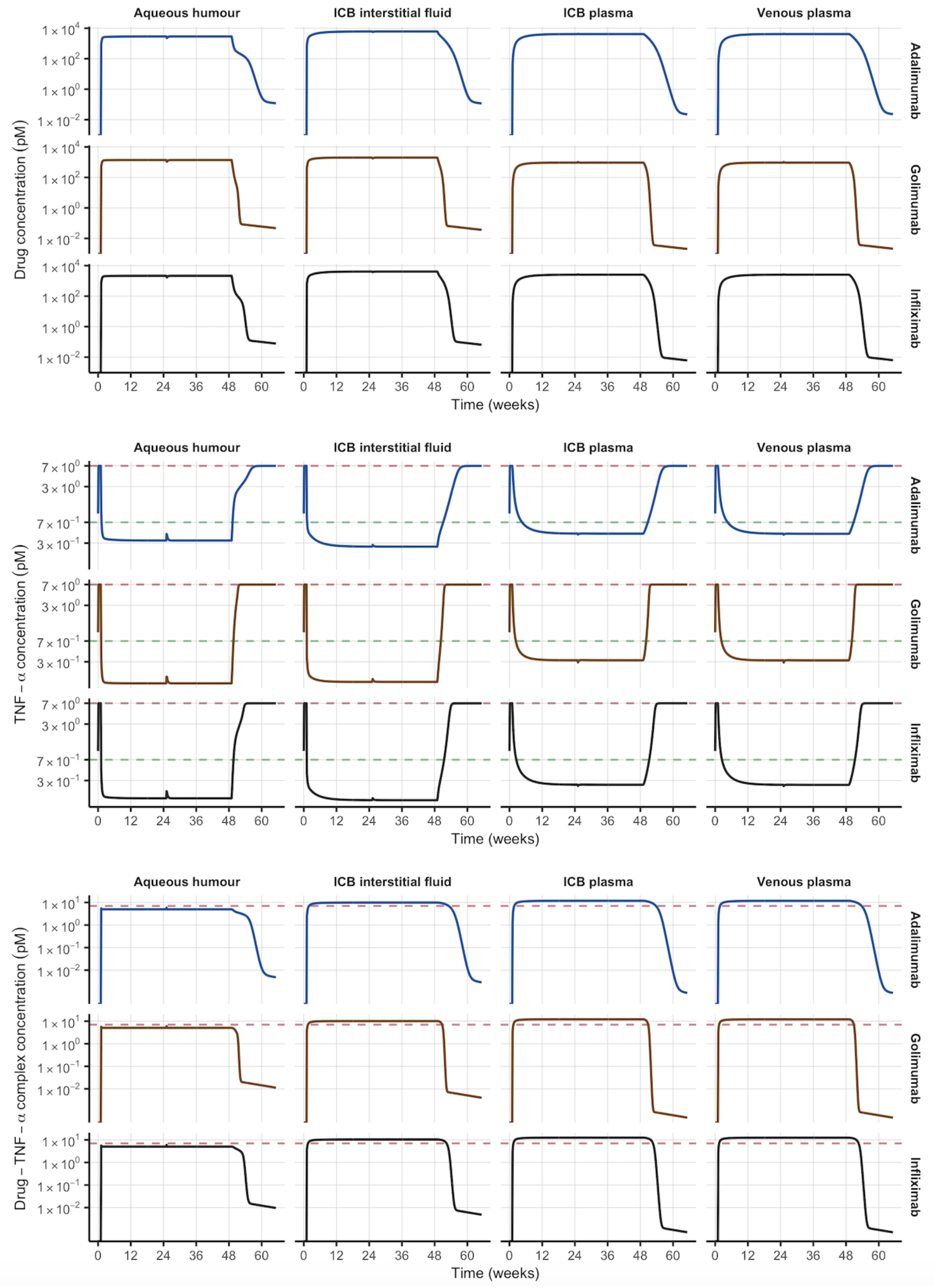

Figure S2. Simulated longitudinal profiles of drug, TNF-α and, drug- TNF-α complex in the anterior chamber (aqueous humour), ICB (interstitial fluid and plasma) and venous plasma over 1 year after the IVT administration of sustained release formulations of different anti-TNF-α mAbs. Red horizontal dashed line: TNF-α pathological concentration (7 pM); green horizontal dashed line: treatment goal (10% of TNF-α pathological concentration). ICB: iris-ciliary body; IVT: intravitreal; mAbs: monoclonal antibodies.

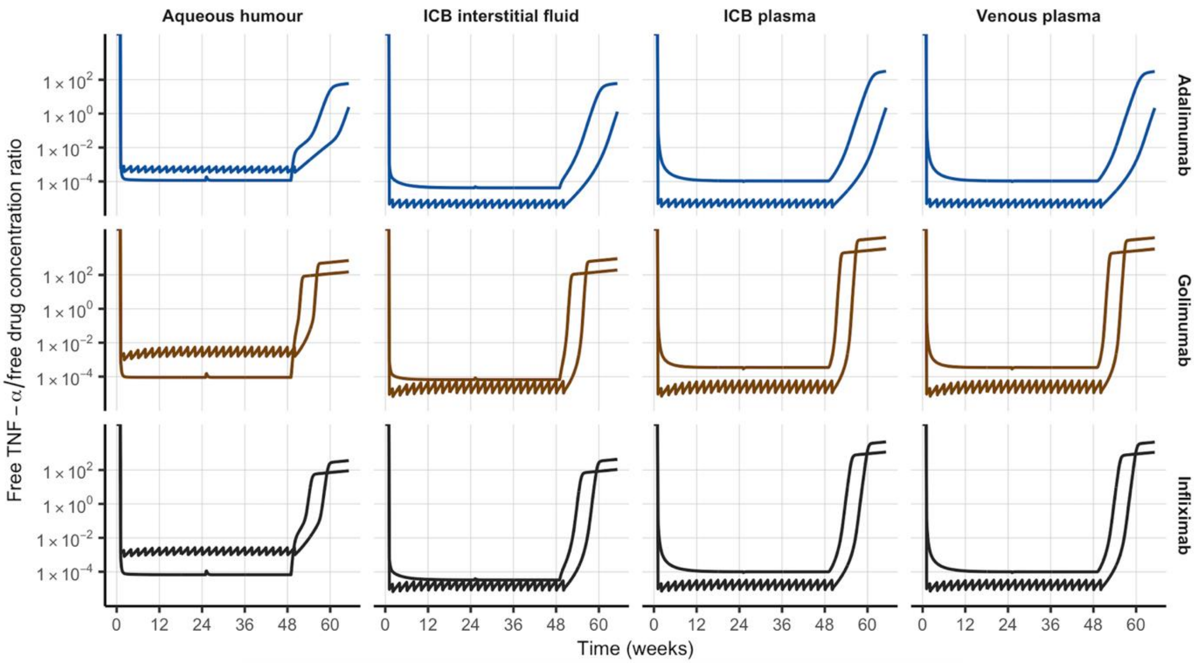

Figure S3. Simulated longitudinal profiles of free TNF-α over free drug concentration ratio in the anterior chamber (aqueous humour), ICB (interstitial fluid and plasma) and venous plasma after the IV administration of reference doses following SOC dosing schedule (sharped profiles) or IVT administration of sustained release formulations Q24W (smooth profiles) of different monoclonal antibodies. ICB: iris-ciliary body; SOC: standard of care; Q24W: every 24 weeks.

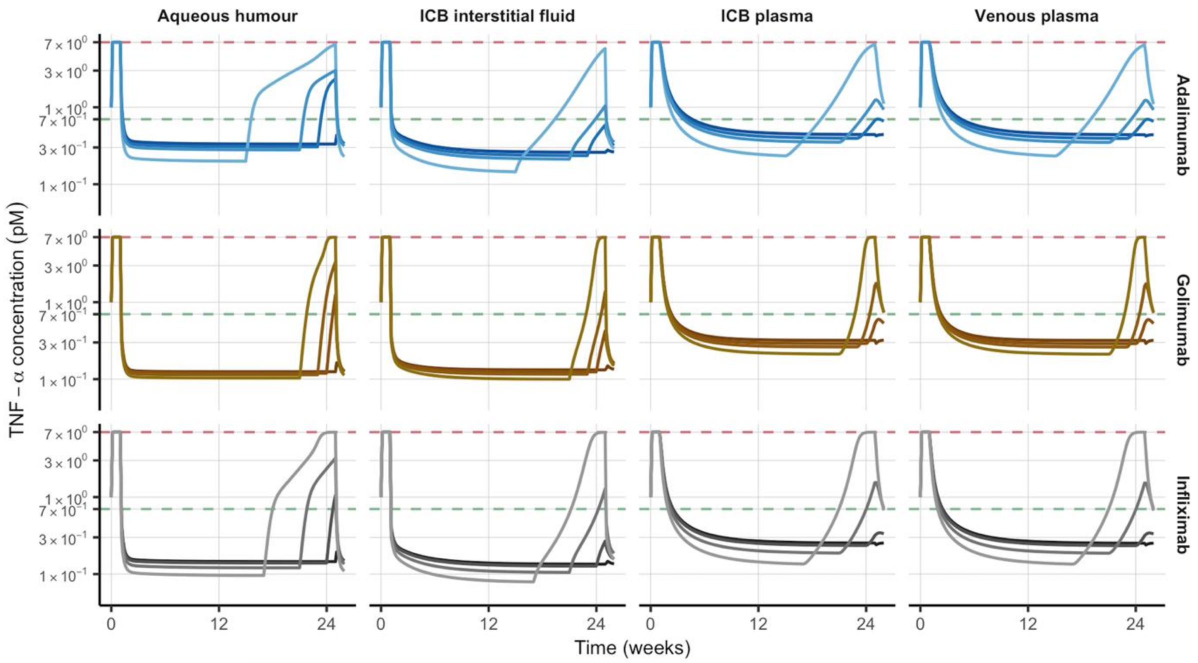

Figure S4. Impact of release rate of anti-TNF-α monoclonal antibodies on TNF-α kinetics in the anterior chamber (aqueous humour), ICB (interstitial fluid and plasma) and venous plasma after the IVT administration of sustained release formulations. Dark profiles correspond to the optimal release rate selected; lighter profiles relate to different degrees of release rate increases (see manuscript Table 4). IVT: intravitreal; ICB: iris-ciliary body.

Local sensitivity analysis

Parameters (P) (inputs): SF, k_{off} and K_D.

Fold change: 2, 4 and 10. Only SF values higher than 1 were assessed in order to avoid scenarios with reduced synthesis compared to the healthy status.

Output (Q): intravitreal release rate of adalimumab, golimumab and infliximab.

Therapeutic objective: ≥ 90% reduction of free TNF-α in aqueous humour, iris-ciliary body (plasma and interstitial fluid) and systemic plasma from pathological levels (i.e., 7 pM). For those scenarios whose SF value was higher than 7 (i.e., 14, 28 and 70), the therapeutic objective was set in absolute terms (free TNF-α ≤ 1 pM) in order to avoid therapeutic objectives well above TNF-α baseline (i.e., 1.4, 2.8 and 7 pM).

Assessment: sensitivity index (SI)

$$SI = \frac{(Q_i - Q_{default})}{(P_i - P_{default})}$$

where Q_i is the new value of model output (Q) corresponding to the new value of input parameter P (P_i) and $Q_{default}$ and $P_{default}$ are the default values of input parameter P and its corresponding model output Q.

Drug	Parameter	Fold-change	Value	Release Rate (μg/week)	Sensitivity Index
**Adalimumab**	SF	0.25	1.75	1.8024	0.0000
**Adalimumab**	SF	0.5	3.5	1.8024	0.0000
**Adalimumab**	SF	1	7	1.8024	NA
**Adalimumab**	SF	2	14	2.4032	0.3333
**Adalimumab**	SF	4	28	6.0079	0.7778
**Adalimumab**	SF	10	70	18.0238	1.0000
**Adalimumab**	k_{off} (h^{-1})	0.1	0.0576	3.1842	-0.8519
**Adalimumab**	k_{off} (h^{-1})	0.25	0.144	1.8024	0.0000
**Adalimumab**	k_{off} (h^{-1})	0.5	0.288	1.8024	0.0000
**Adalimumab**	k_{off} (h^{-1})	1	0.576	1.8024	NA
**Adalimumab**	k_{off} (h^{-1})	2	1.152	1.8024	0.0000
**Adalimumab**	k_{off} (h^{-1})	4	2.304	1.8024	0.0000
**Adalimumab**	k_{off} (h^{-1})	10	5.76	1.8024	0.0000
**Adalimumab**	K_D (μM)	0.1	12.7	1.8024	0.0000
**Adalimumab**	K_D (μM)	0.25	31.75	1.8024	0.0000
**Adalimumab**	K_D (μM)	0.5	63.5	1.8024	0.0000

**Adalimumab**	K_D (μM)	1	127	1.8024	NA
**Adalimumab**	K_D (μM)	2	254	2.1028	0.1667
**Adalimumab**	K_D (μM)	4	508	3.3044	0.2778
**Adalimumab**	K_D (μM)	10	1270	7.8103	0.3704
**Golimumab**	SF	0.25	1.75	0.9796	0.0000
**Golimumab**	SF	0.5	3.5	0.9796	0.0000
**Golimumab**	SF	1	7	0.9796	NA
**Golimumab**	SF	2	14	1.5306	0.5625
**Golimumab**	SF	4	28	3.0613	0.7083
**Golimumab**	SF	10	70	9.1838	0.9306
**Golimumab**	k_{off} (h^{-1})	0.1	0.0335	0.9796	0.0000
**Golimumab**	k_{off} (h^{-1})	0.25	0.084	0.9796	0.0000
**Golimumab**	k_{off} (h^{-1})	0.5	0.168	0.9796	0.0000
**Golimumab**	k_{off} (h^{-1})	1	0.335	0.9796	NA
**Golimumab**	k_{off} (h^{-1})	2	0.67	0.9796	0.0000
**Golimumab**	k_{off} (h^{-1})	4	1.34	0.9796	0.0000
**Golimumab**	k_{off} (h^{-1})	10	3.35	0.9796	0.0000
**Golimumab**	K_D (μM)	0.1	1.8	0.9796	0.0000
**Golimumab**	K_D (μM)	0.25	4.5	0.9796	0.0000
**Golimumab**	K_D (μM)	0.5	9	0.9796	0.0000
**Golimumab**	K_D (μM)	1	18	0.9796	NA
**Golimumab**	K_D (μM)	2	36	0.9796	0.0000
**Golimumab**	K_D (μM)	4	72	1.0408	0.0208
**Golimumab**	K_D (μM)	10	180	1.8368	0.0972
**Infliximab**	SF	0.25	1.75	1.4419	0.0000
**Infliximab**	SF	0.5	3.5	1.4419	0.0000
**Infliximab**	SF	1	7	1.4419	NA
**Infliximab**	SF	2	14	1.6221	0.1250
**Infliximab**	SF	4	28	3.7249	0.5278
**Infliximab**	SF	10	70	10.2135	0.6759
**Infliximab**	k_{off} (h^{-1})	0.1	0.072	1.4419	0.0000
**Infliximab**	k_{off} (h^{-1})	0.25	0.18	1.4419	0.0000
**Infliximab**	k_{off} (h^{-1})	0.5	0.36	1.4419	0.0000
**Infliximab**	k_{off} (h^{-1})	1	0.72	1.4419	NA
**Infliximab**	k_{off} (h^{-1})	2	1.44	1.4419	0.0000
**Infliximab**	k_{off} (h^{-1})	4	2.88	1.4419	0.0000
**Infliximab**	k_{off} (h^{-1})	10	7.2	1.4419	0.0000
**Infliximab**	K_D (μM)	0.1	4.4	1.4419	0.0000

**Infliximab**	K_D (μM)	0.25	11	1.4419	0.0000
**Infliximab**	K_D (μM)	0.5	22	1.4419	0.0000
**Infliximab**	K_D (μM)	1	44	1.4419	NA
**Infliximab**	K_D (μM)	2	88	1.4419	0.0000
**Infliximab**	K_D (μM)	4	176	1.5020	0.0139
**Infliximab**	K_D (μM)	10	440	3.0040	0.1204

TNF-α first order dissociation rate constant (k_{off})

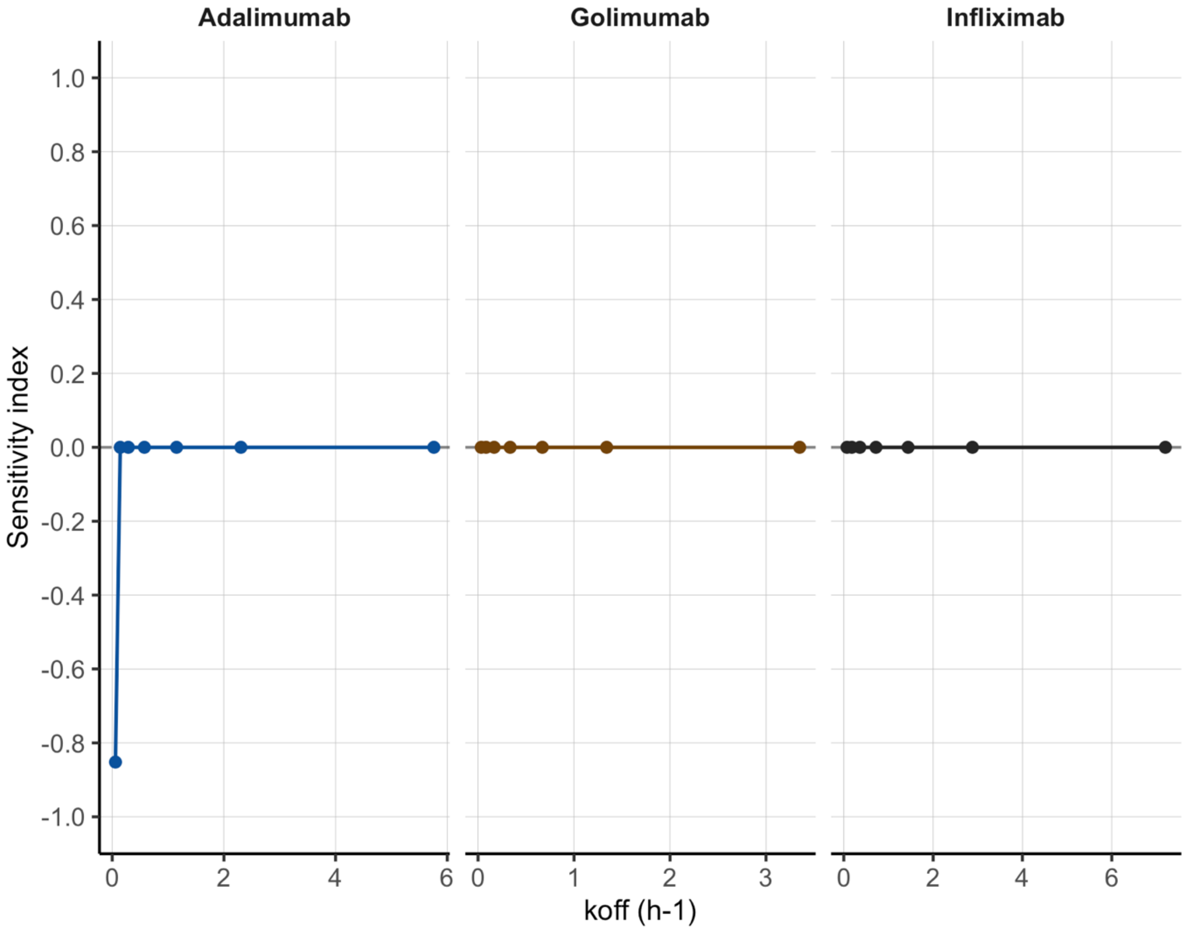

TNF-α dissociation constant (K_D)

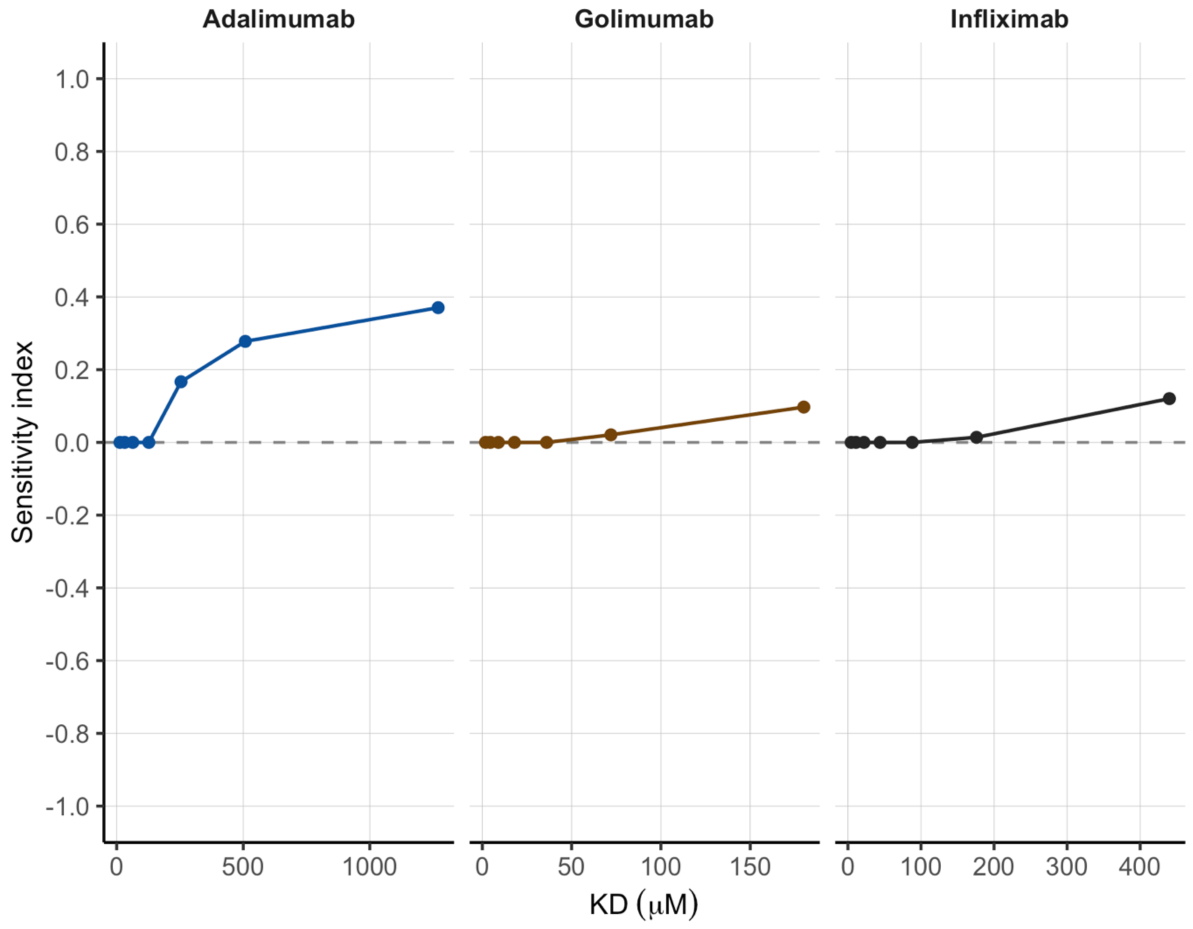

Synthesis factor (SF)

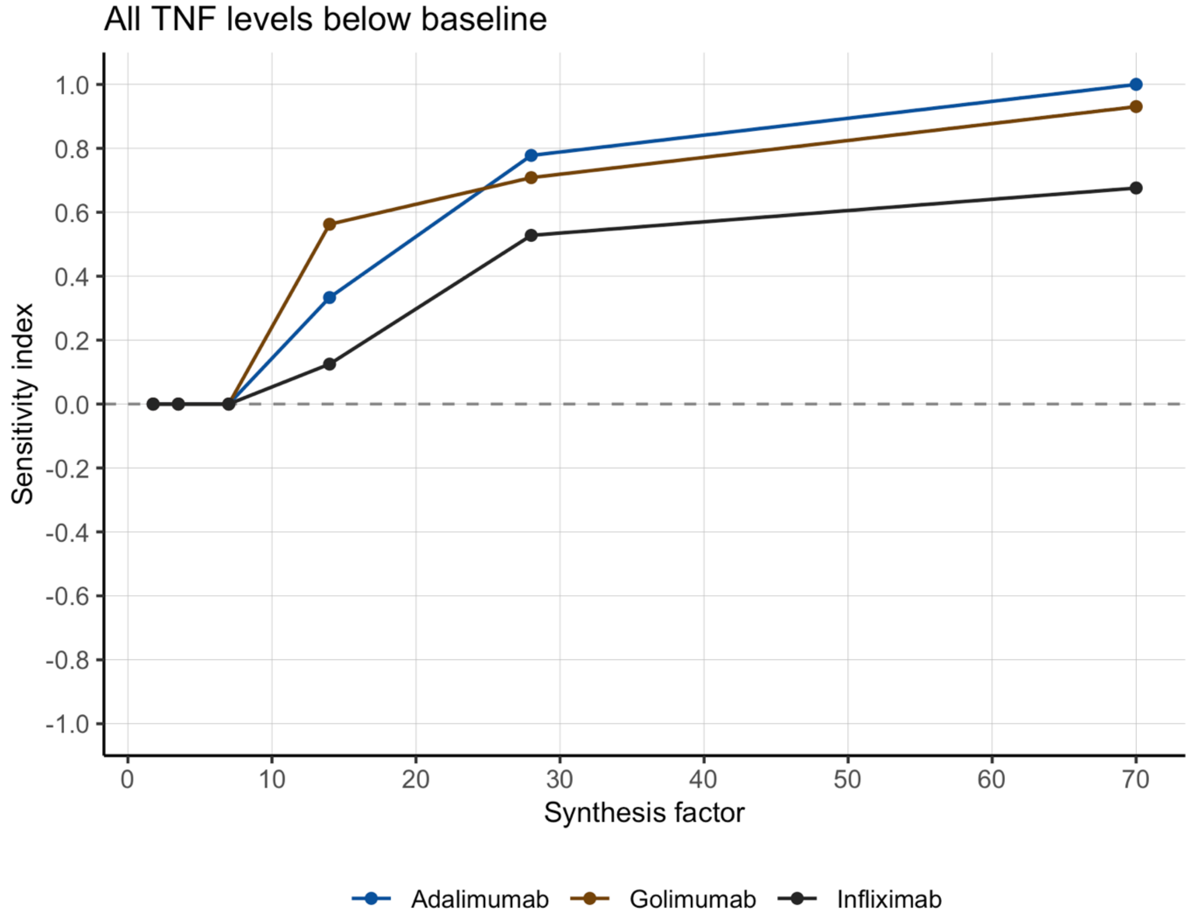

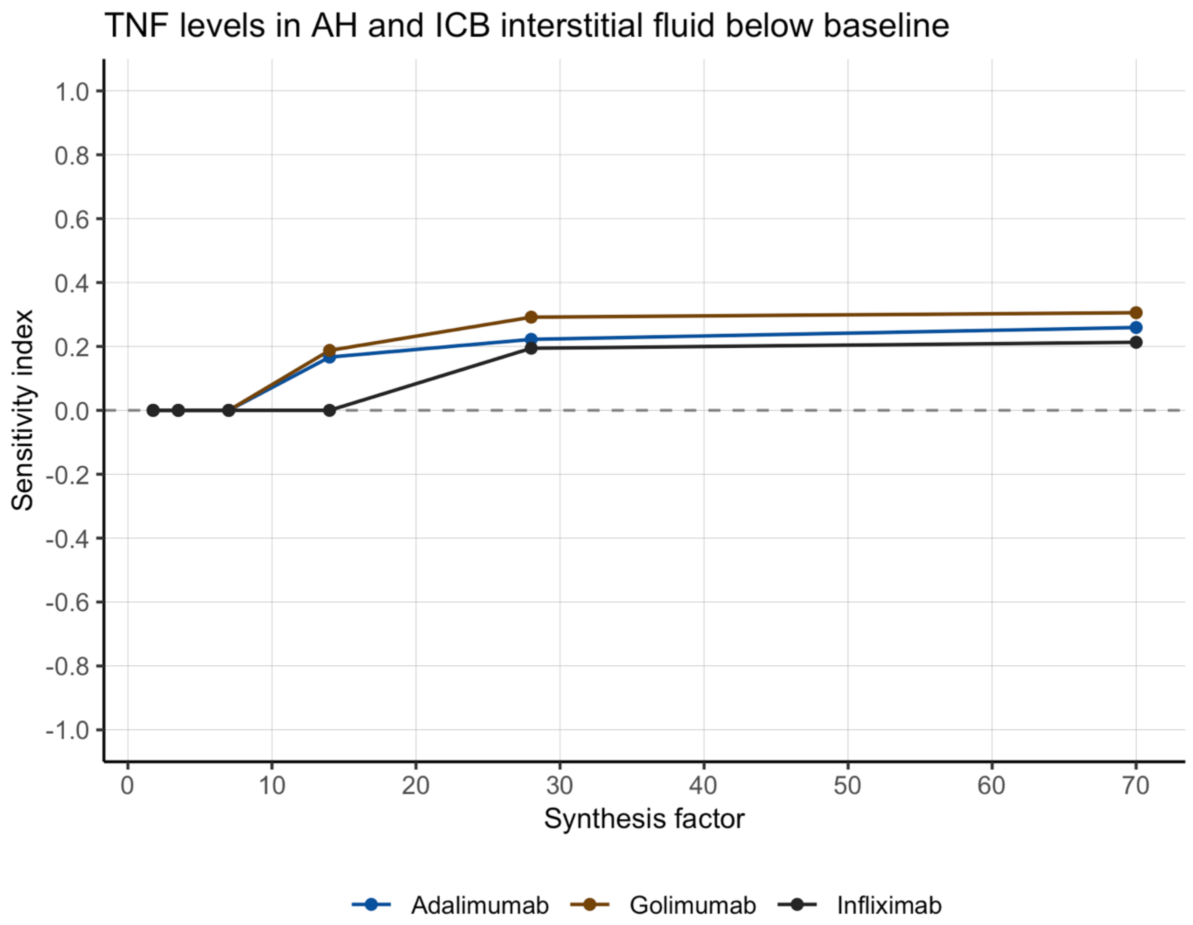

TNF levels in AH and ICB interstitial fluid below baseline
Sensitivity index
1.0
0.8
0.6
0.4
0.2
0.0
-0.2
-0.4
-0.6
-0.8
-1.0
0
10
20
30
40
50
60
70
Synthesis factor
Adalimumab
Golimumab
Infliximab
